# Vestibular Agnosia: Toward a Better Understanding of Its Mechanisms

**DOI:** 10.3390/audiolres15010015

**Published:** 2025-02-11

**Authors:** Assan Mary Cedras, Jonathan Dion, Arnaud Saj, François Champoux, Maxime Maheu

**Affiliations:** 1 School of Speech Language Pathology and Audiology, Montreal University, Montreal, QC H3N 1X7, Canada; assan.mary.cedras@umontreal.ca (A.M.C.); jonathan.dion.3@umontreal.ca (J.D.); francois.champoux@umontreal.ca (F.C.); 2 Institut Universitaire sur la Réadaptation en Déficience Physique de Montréal (IURDPM), Pavillon Laurier, CIUSSS du Centre-Sud-de-l’Île-de-Montréal, Montreal, QC H2H 1C4, Canada; 3 Psychology Department, University of Montreal, Montreal, QC H2V 2S9, Canada; arnaud.saj@umontreal.ca; 4 CRIR/Institut Nazareth et Louis-Braille du CISSS de la Montérégie-Centre, Longueuil, QC J4K 5G4, Canada; 5 Centre Interdisciplinaire de Recherche sur le Cerveau et L’apprentissage (CIRCA), University of Montréal, Montreal, QC H3C 3J7, Canada

**Keywords:** vestibular agnosia, aging, traumatic brain injury, self-motion perception, Parkinson, neurodegenerative disease

## Abstract

**Background/Objectives**: Vestibular agnosia is characterized by a reduced or absent self-motion perception while demonstrating the presence of normal peripheral vestibular function following stimulation. This condition has previously been reported by previous authors in different populations and more recently in traumatic brain injury patients. However, the underlying mechanisms responsible for vestibular agnosia remain a matter of debate. The objective of this manuscript is to review and compare the behavioral and neuroanatomical findings in populations where vestibular agnosia has been demonstrated to better understand the underlying mechanism. **Methods**: A review of the literature was conducted using four databases: Medline, Embase, Google Scholar, and PubMed. A normal vestibulo-ocular reflex function with an impaired self-motion perception following vestibular stimulation represented the inclusion criteria used. **Results**: Behavioral data reviewed in the studies revealed a clear association with postural instability. However, no consensus can be drawn from neuroanatomical data due to variability in brain impairments in those populations even though impairments in the parietal cortex are often reported. **Conclusions**: In general, behavioral data and neuroanatomical data regarding vestibular agnosia have been poorly documented throughout the literature. However, vestibular agnosia can be observed in different populations and is present in concomitant postural control deficits, an important predictor of falls. Finally, even though the parietal cortex has been associated with vestibular agnosia, future studies are required to adequately identify the underlying mechanism. Indeed, the parietal cortex could be part of a larger network mediating vestibular agnosia. This review proposes various methods that future studies should use to overcome the present limitations.

## 1. Introduction

Peripheral vestibular afferents transmit signals related to head acceleration to the brainstem and cerebellum, which then relay information to various reflex pathways. Among these, the vestibulo-ocular reflex (VOR) stabilizes gaze during head movements, while the vestibulo-spinal reflex (VSR) maintains head and body stability in space. Furthermore, the vestibulo-cortical pathway conveys vestibular input to the cerebral cortex, contributing to cognitive processes, spatial body representation, and self-motion perception [1]. At early stages, vestibular signals are integrated with other sensory cues to participate in functions such as self-motion perception. Self-motion perception is the ability to perceive a change in direction or a movement made by our body and is essential for navigation in space. In addition to providing a clear representation of ourselves in space, self-motion perception is of great importance for various daily tasks such as standing balance, walking, and driving [2].

In the past few years, it has been reported that some patients express a reduced or an absence of self-motion perception following vestibular stimulation but demonstrate normal vestibular reflexes [3]. This phenomenon, defined as vestibular agnosia, has also been reported in various populations such as elderly people, traumatic brain injury patients, and patients with neurodegenerative diseases. However, no clear consensus has been made regarding the mechanism regulating vestibular agnosia. A better understanding of the mechanisms responsible for vestibular agnosia is crucial to improve identification and rehabilitation of this condition. In fact, since no objective clinical test is known to evaluate self-motion perception, understanding the mechanism regulating an abnormal self-motion perception constitutes the first step towards the elaboration of an appropriate test and afterward ensures specific vestibular rehabilitation protocol. Therefore, in the present scoping review, we propose to review and compare behavioral and neuroanatomical findings in populations exhibiting vestibular agnosia, with the aim of elucidating identification methods and underlying mechanisms responsible for this diminished or absent vestibular perception despite intact VOR responses.

## 2. Materials and Methods

A review of the literature was conducted according to the PRISMA guidelines provided by Tricco et al. [4]. The literature search was conducted for the period between 1994 to 17 November 2024 using four databases: Medline, Embase, Google Scholar, and PubMed. Search terms included combinations and variations of “vestibular agnosia”, “nystagmus-sensation dissociation”, “absence of vertigo”, “traumatic brain injury”, “elderly”, and “neurodegenerative disease.” Additionally, relevant articles cited in the included studies were reviewed to ensure comprehensive coverage and to avoid missing any eligible studies. The literature search concerned studies in English or French; other languages were excluded. The present scoping review focused on the past 30 years given the advances in imaging and vestibular stimulation methods during this period.

### Study Selection

**Figure 1:**
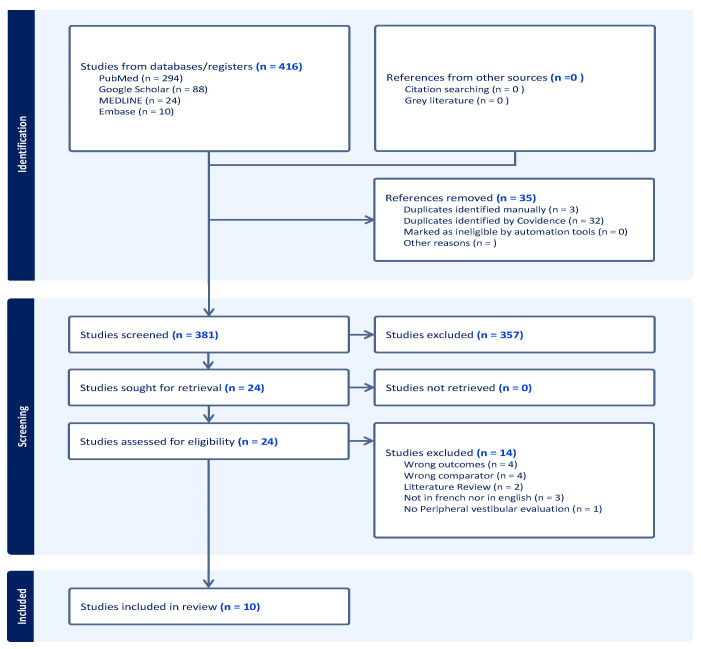
Flow diagram of the study selection process following PRISMA guidelines.

To be included, studies must (1) investigate the absence of self-motion perception, (2) following vestibular stimulation (3) in the presence of an intact peripheral vestibular system, as evidenced by normal vestibulo-ocular reflex (VOR) function. Only those that adequately investigated and isolated vestibular-induced self-motion results were included for studies utilizing multiple methods to induce self-motion perception. Studies involving participants with peripheral vestibular impairments or those that did not assess the VOR function were excluded. Data obtained from the four databases (Medline, Embase, Google Scholar and PubMed) were exported into the web software Covidence. Initially, the first author screened the references based on their abstracts and titles. Secondly, selected articles were read and summarized in an Excel table to enable sorting based on the inclusion criteria mentioned earlier. The second author and last author were consulted to help classify the included studies. Figure 1 illustrates the search flowchart for this review. Afterward, studies were regrouped based on the vestibular-induced self-motion method used.

## 3. Results

In most of the articles included in the literature review, seven used caloric stimulation [5,6,7,8,9,10,11] and three used a rotatory chair [3,12,13] to compare VOR responses and self-motion perception. As these methods differ in the activation of the peripheral vestibular system, the Results Section will discuss the behavioral and neuroanatomical findings of these studies separately. A description of all included articles is provided in Appendix A.

### 3.1. Caloric Stimulation and Self-Motion Perception

Among the seven articles using caloric stimulation, behavioral data were analyzed in all of them, whereas neuroanatomical data were analyzed only in two articles. In the articles where behavioral data were explored, authors assessed self-motion perception using binary responses (by reporting “yes” or “no” if any self-motion perception was perceived, reporting the perceived direction (right or left), or by reporting the type of perceived self-motion (spinning, other sensation, or none) [5,6,7,8,9,10,11]. Finally, to collect neuroanatomical data, studies used single-photon emission computed tomography (CT scan [10,11] and magnetic resonance imagery (MRI) [9,10]).

#### 3.1.1. Behavioral Data

Reduced or absence of self-motion perception following caloric stimulation was reported in different populations such as elderly participants [5,7,8], spinocerebellar degeneration patients [10], cerebrovascular patients [9,11], and patients with dizziness referred for a caloric vestibular evaluation [6].

Studies on elderly participants have shown that patients reporting vestibular symptoms, with mean ages between 66 and 74 years old (74 years [8], 66.55 years [7], and 67.73 years [5]), did not perceive any motion sensation following warm-water or warm-air caloric stimulation even though they demonstrated normal slow-phase eye velocity (SPV > 15°/s), normal video head impulse, and normal vestibular evoked myogenic potential results. In those studies, participants with an absence of self-motion perception were additionally evaluated on other behavioral aspects related to vestibular functions such as their perceived handicap related to dizziness (e.g., Dizziness Handicap Inventory (DHI)) [5,7], static and dynamic postural tasks (e.g., EquiTest) [5,8], and cognitive tests such as visuospatial memory tests called Memory Matches (Lumate, LLC) [5]. Patients with an absence of self-motion following caloric stimulation had greater scores on the DHI compared to their healthy aged-matched controls. On the Equitest, participants who failed to report self-motion perception exhibited greater postural instability during condition 5 (eyes closed with a moving platform) and condition 6 (moving visual surroundings and platform) compared to control groups, who showed normal results. Specifically, 80% of the group without vestibular perception fell during both conditions 5 and 6, while 20% obtained an abnormal score in condition 5 and fell in condition 6 [8].

In the visuospatial memory task (e.g., Memory Matches), where patients had to match eight pairs of cards in the quickest time possible, patients who reported an absence of motion perception had significantly more difficulties completing the task compared to patients with perceived motion perception.

One study reported abnormal self-motion perception in patients with spinocerebellar degeneration using caloric stimulation. Ishibashi et al. [10] asked 179 patients (aged between 20 and 89 years old) with spinocerebellar degeneration to report if they experienced spinning or moving sensations during cold-water caloric stimulation. Among those patients, 21 (11.73%; aged between 50 and 76 years old) did not experience spinning or moving sensations following irrigation even though their maximum SPVs were >15.8°/s. Finally, a case study regarding a 38-year-old patient with cerebral vascular lesions in the bilateral parietal temporal lobes was reported by Kaga et al. [9]. The patient did not report self-motion perception following cold-water caloric stimulation bilaterally, despite normal SPV.

Mijovic et al. [6] reported that 18% of 122 patients with normal caloric and robust SPV reported no self-motion perception. More precisely, 5.7% of 244 ears with a mean SPV of 21°/s reported no self-motion perception following warm caloric stimulation, whereas 8.3% of 59 ears with a mean SPV of 9°/s reported no self-motion perception following cold caloric stimulation. Unfortunately, no demographic data regarding the patients with normal caloric responses and no self-motion perception were reported in the study.

#### 3.1.2. Neuroanatomical Data

Takeda et al. [11], using a CT scan, investigated anatomical findings in two cerebrovascular patients with an absence of vertigo or dizziness following caloric stimulation. They found decreased cortical regional cerebral blood flow in the right parietal temporal lobe. Similarly, the case study reported by Kaga et al. [9] presented lesions in the bilateral parietal temporal lobe. More precisely, using MRI, infarctions were detected in the bilateral primary auditory cortices, as well as in the auditory radiation in the bilateral postcentral gyrus and the partial third frontal gyrus. On the other hand, Ishibashi et al. [10] failed to report common regions in the cortex responsible for the absence of self-motion perception in their cohorts of patients with spinocerebellar degeneration using both CT scan and MRI. In fact, multiple observations in different regions were made, such as atrophy of the pons, atrophy in the temporal and frontal lobes, and reduced up-take in the right thalamus, in the bilateral temporal lobe, and in the bilateral parietal lobe.

### 3.2. Rotatory Chair and Motion Perception

Another method of vestibular stimulation employed in the reviewed studies was the use of a rotatory chair. The group tested for vestibular agnosia using this method consisted of patients with traumatic brain injury (TBI). Vestibular perception using a rotatory chair was assessed in three articles by asking participants to press buttons to identify the direction of rotation (leftward or rightward) [3,12,13]. The rotation velocity of the chair increased progressively with the objective of determining the velocity or acceleration thresholds at which participants could correctly identify the direction of the movement. In two of the studies [3,12], the authors assessed additional behavioral data such as postural stability (via force platform: with eyes open or closed and with or without foam) or subjective vestibular symptoms (via questionnaires such as DHI, ABC questionnaire, and the Patient Health Questionnaire). Neuroanatomical data were assessed in two studies [12,13] using functional structural Magnetic Resonance Imaging (fMRI) or structural and functional MRI.

#### 3.2.1. Behavioral Data

Patients with vestibular agnosia (VA) following TBI had higher vestibular perceptual thresholds than matched healthy controls [3] (Calzolari et al. [3]: mean velocity thresholds of 12.92 ± 14.14°/s for patients compared to 3.87 ± 2.13°/s for controls; Hadi et al. [13]: mean acceleration thresholds of 4.20 ± 3.92°/s^2^ for TBI with vestibular agnosia compared to 0.76 ± 0.42°/s^2^ for controls; Hadi et al. [12]: mean acceleration thresholds for patients with TBI over 1.99°/s^2^ compared to 0.76 ± 0.42°/s^2^ for controls), even though both groups had normal vestibular ocular reflex thresholds (Calzolari et al. [3]: mean SPV of 2.52 ± 2.03°/s for patients and 1.78 ± 1.49°/s for controls; Hadi et al. [12]: mean SPV of 2.52°/s for patient and 1.78°/s for controls). Furthermore, individuals with TBI and vestibular agnosia (VA) demonstrated poorer postural control compared to the control group [3] or exhibited less improvement in balance recovery (smaller gains between baseline and six months post-injury for postural tasks) compared to those with TBI without vestibular agnosia [12]. In both studies, patients with TBI were given the DHI and the ABC scale to assess subjective vestibular symptoms. Additionally, Hadi et al. [12] used the PHQ-9 to assess depression severity. Calzorali et al. [3] failed to reveal significant difference between TBI with and without VA in DHI scores (22.53 ± 17.05 with VA compared to 29.73 ± 22.49 without VA) and between TBI with and without preserved balance in ABC scores (77.19 ± 17.28 versus 72.91 ± 23.81, respectively). Similarly, Hadi et al. [12] found no significant correlation between changes in subjective vestibular symptom scores and their corresponding objective measures. However, they reported that TBI cases with VA had lower mean DHI scores than TBI cases without VA during the acute phase, despite worse results in objective tests. Interestingly, they noted that two TBI cases without VA during the acute phase developed vestibular agnosia between 3 and 6 months following the acute phase. One patient was reported to develop postural instability even if he had normal balance during the acute phase [12]. Another interesting finding in Hadi et al.’s [12] study was that, while the majority of TBI patients showed reduced DHI scores over the follow-up period, a few TBI patients with vestibular agnosia experienced an increase in their DHI scores.

#### 3.2.2. Neuroanatomical Data

Hadi et al. [13] evaluated 11 TBI patients with vestibular agnosia (VA) and 15 TBI patients without VA using the Magnetization Prepared Rapid Gradient Echo (MPRAGE) sequence to acquire 3D T1-weighted images, alongside blood oxygenation level-dependent (BOLD) imaging for functional MRI (fMRI). This approach allowed for the separate assessment of gray- and white-matter regions.

First, the study identified decreased functional connectivity in TBI patients with VA within the bilateral lingual gyri and the right temporo-occipital fusiform cortex, regions associated with visuo-vestibular integration and specifically involved in self-motion processing. Second, white-matter analysis revealed that TBI patients with VA exhibited increased functional connectivity between the left posterior thalamic radiation and the right superior corona radiata, left and right anterior corona radiata, right superior longitudinal fasciculus, and right posterior corona radiata. However, the posterior thalamic radiation showed decreased connectivity with the left uncinate fasciculus. This distinct bilateral and anterior–posterior connectivity pattern suggests its involvement in the neural mechanisms underlying vestibular agnosia.

In a subsequent study, Hadi et al. [12] assessed 17 TBI patients using diffusion tensor imaging to investigate structural brain changes in gray- and white-matter regions over time (e.g., at baseline and six months post-TBI). This study also examined interactions between these structural changes and behavioral measures, including balance control and vestibular perceptual thresholds. Longitudinal analysis revealed significant changes in gray-matter regions. More specifically, the left supplementary motor area, left precuneus, left mid-frontal gyrus, left precentral gyrus, and paracentral lobule revealed significant interactions with behavioral measures, highlighting their relevance to functional recovery. The recovery was specifically associated with changes in the posterior corpus callosal regions, including the splenium, forceps major, and the body of the corpus callosum. These structural changes suggest that recovery of vestibular function may rely on modifications in interhemispheric connectivity mediated by the corpus callosum.

## 4. Discussion

The present review aimed to synthesize and compare behavioral and neuroanatomical data in populations with vestibular agnosia in the presence of normal VOR function with the aim to better understand its mechanism. In summary, an absent or reduced self-motion perception in the presence of normal VOR reflexes was observed in different populations such as TBI patients, elderly patients, spinocerebellar degeneration patients, and in one cerebellar vascular lesion patient. Behavioral data reviewed in the studies revealed a clear association with postural instability. However, no consensus can be drawn from neuroanatomical data due to variability in brain impairments in those populations even though impairments in the parietal, frontal, and temporal cortex as well as the brainstem region are often reported. An absent or reduced self-motion perception following vestibular stimulation with normal reflexes seems to have been studied by only a few studies in the literature. However, the knowledge provided by those studies is crucial and can contribute significantly to vestibular evaluations and form the basis for future studies in the perception field, more precisely in vestibular agnosia. In fact, many aspects of vestibular agnosia remain unknown due to some limitations and matters to take into consideration.

### 4.1. Behavioral Evidence of Vestibular Agnosia

Behavioral data revealed that populations with vestibular agnosia present greater postural instability than patients without vestibular agnosia, specifically during challenging conditions such as in the dark or on an unstable surface. It has been demonstrated that vestibular perception participates in postural control mostly in conditions where other sensory cues, such as somatosensory or visual cues, are challenged [14]. In fact, these authors proposed that the vestibulo-spinal reflex is mainly responsible for stabilizing the body and the head in space for small postural sway but requires cortical mechanisms, like perception and attention, to control larger and faster sway. This is in line with recent evidence that cortical sensorimotor areas contribute to standing balance during challenging conditions [15]. This group studied ongoing cortical oscillations during postural control and proposed that the brain would monitor center of pressure (CoP) velocity and position mostly in conditions where sensory information is altered. Therefore, people with reduced or absence of perception may not efficiently use cortical mechanisms to help maintain balance in challenging sensory conditions, making them more at risk of falling.

From a clinical standpoint, people with vestibular agnosia are less likely to seek vestibular evaluation because they either do not perceive vertigo or feel it less intensely, making it less disruptive to their daily lives. As a result, their vestibular disorders may be diagnosed later than those in people without vestibular agnosia, or they might be misdiagnosed altogether, delaying proper treatment. For example, Benign Paroxysmal Positional Vertigo (BPPV) is the most common peripheral vestibular disorder and a leading cause of imbalance and falls in older adults [16], and it has been reported that vestibular agnosia results in dramatically increased missed BPPV [17]. Without a diagnosis, such patients may remain untreated, putting them at higher risk of falling. It is well known that falling has important impacts on individuals and healthcare systems [18,19] and can lead to long-term functional impairment or even death [20].

Some limitations in the studies assessing behavioral data have been observed. First, seven out of the ten studies reviewed used caloric stimulation to provoke a sensation of self-motion. Most studies simply asked participants a Yes/No question to determine if they felt any movement, while others asked participants to describe the type of sensation or the direction of rotation. These approaches are highly subjective and can vary significantly between individuals. Moreover, the sensation of self-motion during caloric stimulation is not always rotatory and may even differ between ears leading to inconsistent results [6]. Developing a method to objectively quantify self-motion perception during caloric testing could overcome this limitation. It would allow researchers to better understand how perception varies across populations and between individuals and may help reduce variability between studies by uniformizing the outcome. Second, an important limitation in some of the studies regarding behavioral data is that they assessed patients who have been seen in clinics regarding postural instability complaints. Even though the authors stated that they ruled out peripheral vestibular lesions in their cohorts, an exhaustive assessment of the peripheral vestibular system (canals and otolitic organs) was not systematically reported or performed by researchers. Moreover, it remains unknown if these patients could have had a central impairment that could explain poorer balance performance. Finally, studies interested in vestibular function should consider giving access to their raw data which will favor transparency as opposed to studies, including some of the present data, that failed to mention the test used and/or present the results obtained.

Moreover, agnosia is often described as the brain’s inability to process sensory information [21]. Calzolari et al. (2017) were the first, to our knowledge, to link the term to the vestibular system. Similarly to audiological and visual agnosia, where the sensory input is perceived but the identification or discrimination of the stimuli is not achieved, vestibular agnosia could likely be interpreted in the same manner. For example, most of the studies reported in the present review that used rotatory chairs asked their participants to adequately identify the motion direction perceived rather than the presence or absence of movement. However, unlike the studies using a rotatory chair, studies that used caloric stimulation defined vestibular agnosia as the complete absence of motion perception. Even though the inability to identify the motion direction and the absence of motion perception is defined as vestibular agnosia in the literature, both could result from two different mechanisms. Therefore, future studies need to be more precise in defining vestibular agnosia.

### 4.2. Neuroanatomical Evidence of Vestibular Agnosia

In the present review, the parietal cortex was often reported as being impaired. This region is known to integrate, among other sensory information, vestibular information and to create a spatial representation [22,23]. Therefore, the parietal lobe could represent a highly interesting cortical region when it comes to identifying neurophysiological markers of vestibular agnosia. However, impairments in other regions have also been observed in patients with vestibular agnosia. In fact, the frontal lobe, the temporal lobe, and even subcortical regions such as the pons have been associated with vestibular agnosia. This observation could lead to the hypothesis that vestibular agnosia may be mediated by a currently unknown brain network rather than a single area. Future studies should investigate these proposed cortical regions in neurotypical populations following vestibular stimulation while modulating the activity of these regions using brain stimulation techniques such as transcranial direct stimulation (tDCS). This non-invasive technique is often used throughout the literature to electrically alter the function of brain regions. For example, it was found that the use of tDCS to create an imbalance between both parietal regions can alter the VOR, but to our knowledge, no previous studies investigated its influence on self-motion perception [24].

As mentioned above, apart from regions in the cortex itself, anomalies have been reported in the cerebellum and the brainstem region. Regions mentioned in this review such as the pons and the vestibular nuclei are known to be involved in the velocity storage mechanism (VSM) [25]. This mechanism is implicated in the VOR response, postural control, and self-motion perception [26]. Indeed, VSM was proposed to project to cortical areas involved in motion processing such as parietal and temporal areas [27,28]. Therefore, a disruption of higher-order structures could limit the perception of motion, even though VSM is normal (as demonstrated by normal VOR). Since it has already been suggested to monitor VSM in the context of self-motion perception [29], it would be important for future studies to investigate the role of VSM in an abnormal self-motion perception pathology such as vestibular agnosia. Based on the present review, even though a small amount of evidence exists, disruption of the parietal and temporal lobe is a candidate to support vestibular agnosia. Indeed, the parietal cortex integrates multisensory cues and has been demonstrated to be crucial for motion perception [30]. Additionally, this electrical modulation of the parietal cortex has been proposed to modulate slow-phase eye velocity following caloric stimulation in healthy participants [31]. Therefore, a disruption of the temporo-parietal region could disrupt self-motion perception and VOR.

The methods used to acquire neuroanatomical data may also contribute to the observed variability between the studies. The articles retrieved in this review used different imagery techniques such as MRI and CT scan. One important limitation of these brain imagery techniques is that they do not allow one to record during vestibular stimulation and differently activate the cortex, therefore limiting the precision and increasing variability regarding the structures that are directly implicated in vestibular agnosia [31]. Future studies should use techniques that allow vestibular stimulation while recording cortical activity such as Functional Near-Infrared Spectroscopy (fNIRS). This technique presents some advantages compared to MRI, such as being portable, and can be used during head and body movements [32]. Another solution could be to use a stimulation method compatible with MRI such as galvanic vestibular stimulation (GVS). This method could offer interesting insights, as its induced activation and self-motion perception are well-established [33]. More specifically, direct current GVS is well known to induce a sense of rotation around Reid’s plane toward the cathodal side [34]. Therefore, future studies could use GVS and MRI to investigate the central mechanism responsible for self-motion perception.

Finally, within the studies reviewed, data regarding hemispheric dominance were not presented. It is well known that the human central vestibular system is lateralized on the right side for right-handed people and on the left side for left-handed people [23]. However, in the studies reviewed, handedness of participants was not mentioned. This could also contribute to increased variability in the results of the neuroanatomical studies. Future research should consider presenting data regarding the handedness of the participants, as activations between left- and right-handed people following vestibular stimulation are known to be different [23].

## 5. Conclusions

In general, behavioral data and neuroanatomical data regarding vestibular agnosia have been poorly documented throughout the literature. However, vestibular agnosia can be observed in different populations and is present in concomitant postural control deficits, an important predictor of falls. Finally, even though the parietal cortex has been associated with vestibular agnosia, future studies are required to adequately identify the underlying mechanism. Indeed, the parietal cortex could be part of a larger network mediating vestibular agnosia. This review proposes various methods that future studies should use to overcome the present limitations.

## Data Availability

No new data were created or analyzed in this study.

## Appendix A

**Table A1:** Description of the articles retrieved based on the participants’ profile, the vestibular stimulation, and the self-motion assessment.

**Articles**	**Participants**	**Peripheral Vestibular Function Assessment**	**Vestibular Stimulation**	**Self-Motion Assesment**	**Results**
Calzolari et al., 2021 [3]	- 37 acute Traumatic Brain injury (TBI) patients, age = 18–65 - 37 age-matched healthy controls (21 females) mean age = 40.8 SD = 15	Normal vHIT in all TBI patients. - Mean vHIT gain: TBI: 1.04 (SD: ±0.17) (left canal) TBI: 1.04 (SD: ± 0.14) (right canal)	Rotatory chair.	Behavioral: Button (Rightward vs. Leftward)	- TBI patients showed elevated vestibular–perceptual thresholds (TBI 12.92°/s versus control 3.87°/s) but normal vestibular–ocular reflex thresholds (TBI 2.52°/s versus control 1.78°/s). - TBI patients with elevated vestibular–perceptual thresholds (+3 SD above controls’ average) were designated as having vestibular agnosia. - TBI patients with vestibular agnosia showed worse posturography than non-vestibular agnosia patients, despite no difference in vestibular symptom scores.
Chiarovano et al., 2016 [8]	- 10 patients with complaints of postural instability (PI) (mean age = 77 years, SD = 8, age range66–85years) - 10 control sex- and age-matched individuals without PI complaints (mean age = 74 years, SD = 6, age range 67–85 years)	Normal HvHIT, cVEMP and oVEMP in all participants. - Mean HvHIT gain: PI: 0.95 ± 0.11(left canal) Control: 0.90 ± 0.08(left canal) PI: 1.02 ± 0.07 (right canal) Control: 1.02 ± 0.06 (right canal) - cVEMP corrected amplitude (μV) and latency (ms): PI: 0.41 ± 0.67; 15.3 ± 0.9 (p1); 15.0 ± 1.1 (n1). Control: 0.56 ± 0.45; 15.0 ± 1.1 (p1); 21.4 ± 1.5 (n1) - oVEMP corrected amplitude (μV) and latency (ms): PI: 2.1 ± 4.0; 11.4 ± 0.5 (n1); 15.4 ± 1.0 (p1). Control: 5.9 ± 5.3; 11.0 ± 0.4 (n1); 14.6 ± 0.8 (p1).	Caloric test	Behavioral: Feeling of body rotation clearly identified to the right or the left (Yes/No)	- Both groups had normal SPV (>15°/s), but PI group had no perception of rotation, and all controls had perception of rotation - SOT on the EquiTest: Control: normal PI: 80% fell in conditions 5 and 6; 20% had a low score in condition 5 and fell in condition 6
Hadi et al., 2022 [13]	- 11 Traumatic Brain Injury (TBI) patients with vestibular agnosia (VA+) (4F). mean age = 43, SD = 15.34 - 15 Traumatic Brain Injury (TBI) patients without vestibular agnosia (VA-) (2F) mean age = 42.07, SD = 13.61	Normal vHIT in all TBI patients. - Mean vHIT gain: TBI: 1.04 (SD: ±0.17) (left canal) TBI: 1.04 (SD: ± 0.14) (right canal)	Rotatory Chair	Neuroanatomical: Functional Magnetic Resonance Imaging (MRI)	Vestibular agnosia is linked to the following: Altered functional connectivity in a bilateral white-matter network (left and right anterior corona radiata (ACR), right superior corona radiata (SCR), right superior longitudinal fasciculus (SLF), right posterior corona radiata (PCR), left uncinate fasciculus (UNC), and left posterior thalamic radiation (PTR)). A decreased functional connectivity in bilateral lingual gyri (V3v/V4) and temporo-occipital regions.
Hadi et al., 2024 [12]	- 39 TBI patients (VA+ et VA-) with preserved peripheral vestibular function (mean age = 41.64 years, SD = 13, age range18–65 years). - 37 age- and sex-matched healthy controls (mean age = 40.78 years, SD = 14.75)	Normal vHIT in all TBI patients. - Mean vHIT gain: TBI: 1.04 (SD: ±0.17) (left canal) TBI: 1.04 (SD: ± 0.14) (right canal)	Rotatory chair	Behavioral: Button (Rightward vs. Leftward)	- At 6 months, VA+ but not VA− patients had worse balance compared to controls (no difference between VA+ and VA−). - VA+ had worse balance recovery than VA−. - Two acute VA− patients developed vestibular agnosia at the 6-month follow-up, one of whom also developed imbalance despite having normal balance acutely.
				Neuroanatomical: Structural and Functional Magnetic Resonance Imaging (MRI) (17 TBI patients)	Longitudinal change in sway and vestibular perception thresholds was linked with: - Change in gray matter in the left supplementary motor area, left precuneus, and the left mid-frontal gyrus - Change in white matter localized primarily in posterior corpus callosal regions, including the splenium of the corpus callosum, forceps major, and body of the corpus callosum.
Ishibashi et al., 2003 [10]	- 179 spinocerebellar degeneration patients (SCD) (91 males, 88 females; age range 20–89 years)	Normal peripheral vestibular function in all SCD patients (tests not specified except from caloric test)	Caloric test	Behavioral: Experience of spinning or moving sensations (yes/no)	- Behavioral: 21 patients (11.7%; 39 sides; 15 males, 6 females; age range 55–76 years) did not experience spinning or moving sensations during testing although their maximum SPEVs were >15.8°/s.
				Neuroanatomical: Computed tomography scan (CT scan)—magnetic resonance imagery (IRM)	Neuroanatomical: Common lesions in the cerebral cortex could not be detected using either MRI or single-photon emission CT.
Jacobson et al., 2018 [7]	- 92 patients (mean age = 59.18 years, SD = 18.2) who were referred for an assessment of their dizziness.	No other vestibular tests were reported except for caloric stimulation (normal).	Caloric test (only warm irrigation)	Behavioral: Sensation of movement (Yes/No)	- VA+ had a mean age of 66.55 years and VA- had a mean age of 56.51 years (normal SPV for all participants in both ears and no significant ear effect on vertigo perception). - maxSPV and age are significant predictor for vertigo perception. For every + 1 year, the odds of an absence of vertigo perception are increased 6% per year. For every + 1 deg/sec maxSPV, the odds of an absent perception of vertigo are decreased by 10%.
Kaga et al., 2015 [9]	- A right-handed 38-year-old man with cerebral vascular lesions in bilateral auditory cortices.	Normal rotation nystagmus in the damped rotational chair test and caloric test.	Caloric test	Behavioral: Experience of spinning or moving sensations (yes/no)	Behavioral: the patient reported not experiencing any vestibular sensation at all, despite normal reaction in both ears.
				Neuroanatomical: Magnetic resonance imagery (MRI)	Neuroanatomical: Presence of lesions: The bilateral primary auditory cortices Auditory radiation In the bilateral postcentral gyrus The partial third frontal gyrus
Mijovic et al., 2018 [6]	161 adult patients referred for a VNG by an ENT doctor (60 men and 101 women; mean age = 53.9 years, SD = 14.9); 39 patients were excluded (no VNG or self-motion documented)	No other vestibular tests were reported except for caloric stimulation (normal)	Caloric test (all had warm caloric stimulation; 72 had cold and 39 had unilateral weakness identified)	Behavioral: Sensation of movement (Spinning/rotating, Other, No sensation)	- Warm: 5.7% of 244 ears, with a normal SPV, caused no motion perception (SPV mean = 21°/s) (18.0% had no spinning sensation) - Cold: 8.3% of 59 ears, with a normal SPV, caused no motion perception (SPV mean = 9°/s) (25.0% had no spinning sensation)
Piker et al., 2020 [5]	- VA+: 11 patients without motion perception during warm caloric stimulation (mean age = 67.73 years, SD = 8.4). - VA−: 14 patients with motion perception during warm caloric stimulation (mean age = 50.64 years, SD = 15.2). - 15 age-matched healthy controls (mean age = 60.1 years, SD = 5.9)	Normal vHIT, oVEMP and cVEMP in all participants - Mean vHIT gain: VA+: 1.05 ± 0.13 VA−: 1.05 ± 0.20 - cVEMP amplitude (μV); latency (ms); interaural amplitude asymmetry (%): VA+: 206.9 ± 103.8; 15.2 ± 1.0; 15.1 ± 9.0 VA−: 176.4 ± 86.8; 15.0 ± 0.85; 15.5 ± 8.9 - oVEMP amplitude (μV); latency (ms); interaural amplitude asymmetry (%): VA+: 16.9 ± 14.9; 10.8 ± 0.54; 18.4 ± 13.4 VA−: 13.9 ± 9.4; 10.7 ± 1.0; 16.9 ± 11.7	Caloric test (only warm irrigation)	Behavioral: Sensation of movement (Yes/No)	- VA− group was significatly younger than VA+ group. - VA+ group was significantly poorer than VA- group in Memory Match task and during SOT condition 5 (only conditions 1-2-4-5 of the SOT were performed) - No difference in the DHI score between VA+ and VA− groups (mean VA+: 33.8 ± 24.9; VA−: 38.5 ± 18.7)
Takeda et al., 1995 [11]	- 2 cerebrovascular patients with nystagmus-sensation dissociation. (Patient A: 74 years old; Patient B: 84 years old)	No other peripheral vestibular tests were reported except from caloric stimulation (normal)	Caloric test	Behavioral: Any sensation of motion (yes/no)	Behavioral: Absence of vertigo or dizziness.
				Neuroanatomical: Single-photon emission computed tomography.	Decreased cortical regional cerebral blood flow (rCBF) in the right parietal–temporal lobe.

TBI: Traumatic brain injury; VA: vestibular Agnosia (+ = with, − = without); SOT: Sensory Organization Test (condition 5: the support base moved adaptively following subject’s movements while eyes were closed; condition 6: the support base and the cabin moved in a synchronized way); vHIT: video head impulse test; cVEMP: cervical vestibular evoked myogenic potential; oVEMP: ocular vestibular evoked myogenic potential; HvHIT: horizontal video head impulse test.
